# Reproductive success of three passerine species exposed to dioxin-like compounds near Midland, Michigan, USA

**DOI:** 10.1007/s10646-012-0869-4

**Published:** 2012-03-06

**Authors:** Timothy B. Fredricks, Matthew J. Zwiernik, Rita M. Seston, Sarah J. Coefield, Cassandra N. Glaspie, Dustin L. Tazelaar, Denise P. Kay, John L. Newsted, John P. Giesy

**Affiliations:** 1 Department of Zoology, Michigan State University, East Lansing, MI 48824 USA; 2 Department of Animal Science, Michigan State University, East Lansing, MI 48824 USA; 3 Cardno ENTRIX, Inc., Okemos, MI 48864 USA; 4 Department of Veterinary Biomedical Sciences and Toxicology, Centre University of Saskatchewan, Saskatoon, SK S7J 5B3 Canada; 5 Department of Biology and Chemistry and State Key Laboratory for Marine Pollution, City University of Hong Kong, Kowloon, Hong Kong SAR China; 6 Present Address: Monsanto Company, 800 North Lindbergh Blvd., O3A, St. Louis, MO 63167 USA

**Keywords:** Tittabawassee River, Furans, Dioxins, Passerines

## Abstract

Nests of three passerine birds, house wren (HOWR), tree swallow (TRES), and eastern bluebird (EABL) were monitored daily (2005–2007) at study areas (SAs) downstream of Midland, Michigan where soil and sediment concentrations of polychlorinated dibenzofurans (PCDFs) were significantly greater than the regional background concentrations and upstream reference areas (RAs). Similarly, TRES research conducted at sites contaminated with dioxin-like compounds indicated that concentrations of polychlorinated dibenzo-*p*-dioxins and PCDFs, expressed as ΣPCDD/DFs and 2,3,7,8-tetrachlorodibenzo-*p*-dioxin equivalents observed in the diet and eggs of these three species would be predicted to cause significant effects on reproduction. However, site-specific reproductive parameters including hatching success and fledging success at downstream SAs were similar to or greater than those at upstream RAs. Specifically, hatching success was not significantly different among years, species, locations, or between early and late nesting attempts. Of all initiated clutches, 66% (*n* = 427), 73% (*n* = 245), and 64% (*n* = 122) successfully fledged at least one nestling for HOWR, TRES, and EABL, respectively. Overall reproductive performance was similar between SAs and RAs. The reason for these unexpected results is consistent with the fact that there are species-specific and congener-specific differences in sensitivities to the effects of aryl hydrocarbon receptor agonists.

**Electronic supplementary material:**

The online version of this article (doi:10.1007/s10646-012-0869-4) contains supplementary material, which is available to authorized users.

## Introduction

Portions of the Tittabawassee and Saginaw rivers and associated floodplains downstream of Midland, Michigan USA have concentrations of polychlorinated dibenzofurans (PCDFs) and polychlorinated dibenzo-*p*-dioxins (PCDDs) in soils and sediments that are greater than the background concentrations for the region (Hilscherova et al. 2003). The sum concentrations of the 17 individual 2,3,7,8-substituted PCDD/DFs (ΣPCDD/DFs) in floodplain soils and sediments from the study area (SA) downstream of Midland, Michigan ranged from 1.0 × 10^2^ to 5.4 × 10^4^ ng/kg dry weight (dw), respectively, while mean ΣPCDD/DF concentrations in soils and sediments upstream of Midland were 10- to 20-fold less (Hilscherova et al. 2003). The mixture of chlorinated hydrocarbons in the SA is dominated by a few PCDF congeners, which makes this site distinct from most other locations that are contaminated with polychlorinated biphenyls (PCBs) or PCDDs (Custer et al. 2005; Froese et al. 1998; Harris and Elliott 2000; Neigh et al. 2006a, 2007).

The primary toxicological responses to dioxin-like compounds are mediated through the aryl hydrocarbon receptor (AhR) and effects include carcinogenicity, immuno-toxicity, and adverse effects on reproduction, development, and endocrine functions (van den Berg et al. 1998) and decreased hatching success, adult attentiveness, and immune function of passerine birds (Custer et al. 2005; Martinovic et al. 2003). Recent findings suggest the magnitude of response varies among species of birds (Karchner et al. 2006).

Three cavity-nesting passerines in the vicinity of the Tittabawassee and Saginaw Rivers and the associated floodplain downstream of Midland, Michigan were studied by use of a multiple lines of evidence approach. Tree swallows (TRES; *Tachycineta bicolor*), which eat primarily emergent aquatic invertebrates, are linked to contaminated sediments (Echols et al. 2004; Neigh et al. 2006a), and have been extensively utilized in field studies (Custer et al. 2005; Echols et al. 2004; Froese et al. 1998). House wrens (HOWR; *Troglodytes aedon*) and eastern bluebirds (EABL; *Sialia sialis*) eat primarily terrestrial insects and have been used to assess contaminated soils (Mayne et al. 2004; Neigh et al. 2006a, 2007).

Concurrent studies quantified concentrations of PCDD/DFs and 2,3,7,8-tetrachlorodibenzo-*p*-dioxin equivalents (TEQ_WHO-Avian_) based on World Health Organization toxic equivalence factors (TEF_WHO-Avian_) (van den Berg et al. 1998) in dietary items, reconstituted diet, eggs, and nestlings of HOWR, TRES, and EABL (Fredricks et al. 2011a, d). Accumulation of ΣPCDD/DFs and TEQ_WHO-Avian_ from site-specific dietary insects by nestlings at SAs was 31- to 121-fold and 9- to 64-fold greater than at proximally located reference areas (RAs), respectively. The sum of concentrations of PCDD/DFs in eggs of HOWR and EABL from SAs were 4- to 19-fold greater compared to those from RAs, while eggs of TRES were similar among areas (see Fredricks et al. 2011d for an in-depth discussion of these results). Concentrations of ΣPCDD/DFs in nestlings of all studied species at SAs were dominated by 2,3,7,8-tetrachlorodibenzofuran (TCDF) and 2,3,4,7,8-pentachlorodibenzofuran (2,3,4,7,8-PeCDF) and 4- to 49-fold greater compared to RAs. Concentrations of TEQ_WHO-Avian_ in both eggs and nestlings of all three species were positively correlated with concentrations of ΣPCDD/DFs (Fredricks et al. 2011d). Hatching success for TRES was weakly negatively correlated with concentrations of TEQ_WHO-Avian_ in individual eggs (Fredricks et al. 2011c).

The primary objectives of the study described herein were to assess the site-specific reproductive success of three insectivorous passerines exposed to PCDD/DFs through both terrestrial and aquatic pathways. It was predicted, based on site-specific soil and sediment concentrations that the reproductive performance of these passerines would be negatively affected at downstream SAs compared to upstream RAs. Incorporating an extensive study of nesting success with additional lines of evidence including information on concentrations of residues of concern in eggs and juvenile birds (Fredricks et al. 2011d) and in the diet (Fredricks et al. 2011a, c) allowed for a comprehensive test of the risk assessment process.

## Experimental section

### Site description

**Fig. 1:**
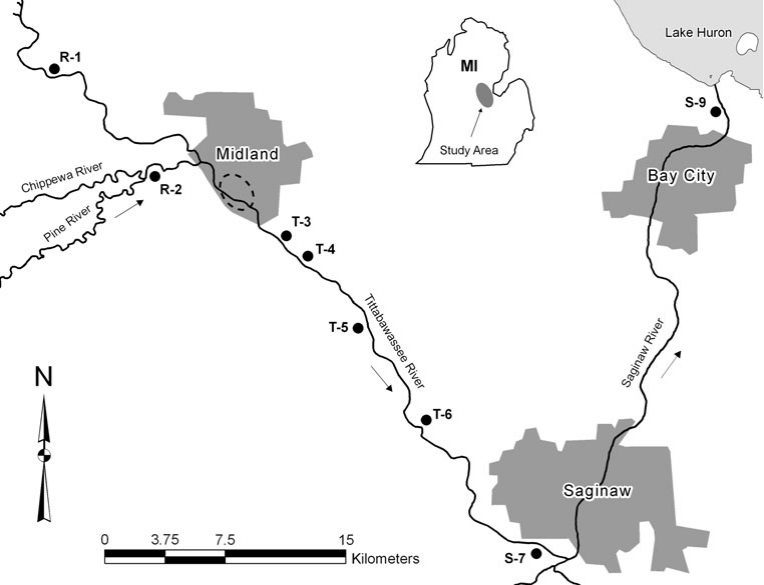
Study site locations within the Chippewa, Tittabawassee, and Saginaw River floodplains, Michigan, USA. Reference Areas (*R-1*–*R-2*), Tittabawassee River Study Areas (*T-3*–*T-6*), and Saginaw River Study Areas (*S-7* and *S-9*) were monitored from 2005 to 2007. Direction of river flow *arrows*; suspected source of contamination *dashed oval*

All study locations were on the Tittabawassee, Chippewa, and Saginaw Rivers in the vicinity of Midland, Michigan (Fig. 1). Nest boxes were located within the 100-year floodplains. Two RAs were upstream of the putative sources of PCDD/DFs (Hilscherova et al. 2003) on the Tittabawassee (R-1) and Chippewa (R-2) rivers (Fig. 1). Study areas downstream of the putative sources of PCDD/DFs include approximately 72 km of free flowing river below the low-head dam near Midland, Michigan, to the confluence of the Tittabawassee and Saginaw Rivers and to where the Saginaw River enters Saginaw Bay. The SAs within the Tittabawassee River area included four locations (T-3–T-6) approximately equally spaced, and two locations (S-7 and S-9) which are approximately at the initiation and terminus of the Saginaw River (Hilscherova et al. 2003). Individual nest box trails within RAs and SAs each contained between 30 and 60 nest boxes that spanned a continuous foraging area of between 1 and 3 km.

### Nest box monitoring

Standard passerine nest boxes (Fredricks et al. 2011d) with wire mesh predator guards around the entrance hole were mounted to a greased metal post and placed at RAs and Tittabawassee River SAs in 2004 and the Saginaw River SAs in 2005. Boxes were placed in species-specific micro-habitats at each site to concurrently maximize occupancy at study sites by HOWR, TRES, and EABL. Boxes were monitored daily after clutch initiation (day first egg laid) through incubation (eggs warm to the touch through hatch day) and then near the expected hatch day for each species. Eggs were individually numbered with indelible ink and massed on a pan balance (±0.001 g) on the date laid. Clutches were considered abandoned if there was no adult activity for 7 days, or new nesting material was added over cold eggs. Hatch day (brood day 0) was determined as the day when the majority (≥50%) of the eggs in a clutch had hatched. The nesting period was considered the time from hatch day to when the nestlings fledged from the nest box (typically 14–20 days depending on the species).

Eggs were collected to measure concentrations of residues. There are two approaches to estimating exposure. One is to take eggs from paired clutches and the other is to collect eggs from separate clutches. Each method has advantages and disadvantages. It was decided that the most accurate estimate of exposure was to collect paired eggs (a single egg per clutch) after clutch size was determined. Therefore, clutch size was not adjusted for egg sampling. However, hatching success, fledging success, and productivity measurements were calculated based on an adjusted clutch size since the fertility and hatchability of the collected egg was unknown at collection. The proportion of clutches from which eggs were removed for residue analyses were 6–25, 17–32, and 3–23%, for HOWR, TRES, and EABL, respectively. Since the outcome that would have occurred was unknown for a sampled egg, an adjusted clutch size was defined as the clutch size excluding any eggs sampled or broken by researchers. Hatching success was defined as the number of eggs that hatch per adjusted clutch size. Brood size and number of fledglings were predicted based on the adjusted hatching success and productivity, respectively. Since fully developed nestlings were collected (a single nestling per brood) just prior to fledge, it was assumed that any nestlings collected would have successfully fledged provided the remaining portion of the nesting attempt was successful. Fledging success was defined as the number of nestlings that fledge per number of eggs that hatched. Productivity was defined as the number of nestlings that fledge per adjusted clutch size. Measures of nesting success for all clutches were included in comparisons up to the point that they were preyed upon, abandoned due to human interference or failed and thereafter removed from comparisons.

To account for some of the potential differences between first and subsequent nesting attempts as well as any temporal variation within seasons, nesting attempts were characterized as occurring ENS (early nesting season) or LNS (late nesting season) during the nesting season. Species-specific mean dates of clutch incubation (23 June, 24 May, and 29 May for HOWR, TRES, and EABL, respectively) were used to separate the nesting season into two parts and to limit the data set to include only those clutches that were completed (Fig. S1). Reproductive success of individually banded females was reported by nesting season and averaged over the whole study.

Masses of individual nestlings were determined multiple times for each brood during 2005 and 2006 for HOWR and TRES, while EABL were also measured in 2007. Masses of HOWR were measured 3, 6, 9, and 10 days post-hatch, while masses of TRES and EABL were determined 4, 8, 12, and 14 days post-hatch. Mass gained per day was calculated as the third minus the first mass measurement divided by the days between. Logistic growth curves were fit to nestling masses over the nesting period (Kaiser and Lindell 2007), and growth rate constants were estimated using site-specific asymptotic values based on nestling masses [HOWR, 12 g (*n* = 205 broods); TRES, 28 g (*n* = 133 broods); EABL, 32 g (*n* = 76 broods)]. Since the initial mass on brood day 0 was not measured, the mean egg mass by species was used. Nestlings were collected for measurement of residues either 10 days post-hatch for HOWR or 14 days post-hatch for TRES and EABL.

Nestlings and adults of HOWR, TRES and EABL were banded with US Fish and Wildlife Service aluminum leg bands throughout the study. Nestlings were banded on the first day that mass was determined for all species except if HOWR nestlings were less than 5 g in which case they were banded when sufficiently developed. Adults were opportunistically captured during routine box monitoring, or actively trapped by researchers during brood rearing. Gender and age of adults were determined following methods outlined in Pyle (1997). During routine handling nestlings and adults were monitored for gross external morphological abnormalities.

Throughout the study the majority of adult females were individually identified during each nesting attempt. This enabled comparisons to be made on an individual, per season basis as well as overall measures of nesting success by individual females for the study period. However, females that were unsuccessful in hatching clutches often could not be banded (16% of HOWR; 6% of TRES; 7% of EABL) which likely resulted in an underestimation of both seasonal as well as overall number of nesting or re-nesting females. Nesting attempts that were preyed upon, abandoned, or otherwise unsuccessful were still included in a female’s yearly and overall measures of nesting success.

### Statistical analyses

Statistical analyses were performed using SAS^®^ software (Release 9.1; SAS Institute Inc., Cary, NC, USA). The experimental unit for measurements associated with eggs, nestlings, and nest success was the nest box, since individual measurements within a clutch cannot be considered independent. Similarly, measures of nest success were reported per nesting attempt, thus making each attempt per box a separate experimental unit (Pinkowski 1979). Based on the frequency of monitoring and use of defined nesting locations (boxes) the authors decided not to use any statistical methods designed to model avian nest survival. Prior to the use of parametric statistical procedures, normality was evaluated using the Shapiro–Wilk’s test and the assumption of homogeneity of variance was evaluated using Levene’s test. Nest parameters that were not normally distributed were ranked prior to statistical analyses. PROC GLM was used for comparisons and when significant differences among locations were indicated, Bonferroni’s *t* test was used to compare individual locations. PROC NLIN was used to fit growth curves based on nestling masses. Differences were considered to be statistically significant at *p* < 0.05.

## Results

### Nesting success

Adult HOWR, TRES, and EABL initiated nests at each study site with the exception of S-9 in which no EABL nested. From 2005 to 2007 at all study sites 427, 245, and 122 clutches were initiated by HOWR, TRES, and EABL, respectively (Table S1). Of all initiated clutches, 65, 72, and 61% successfully fledged at least one nestling for HOWR, TRES and EABL, respectively. Clutch initiations were distributed among study sites such that most sites had approximately 10% of the overall nesting attempts for each species. Nests that were preyed upon or abandoned comprised the majority of nests that were not successful (Table S1). Predation events were categorized by criteria defined by Etterson et al. (2007), and for all studied species were primarily from competition with other studied species for a nest box or due to predation by mammals. Snakes only preyed upon nests at S-7. Nest abandonment and predation events accounted for approximately 25, 23, and 27% of HOWR, TRES, and EABL clutches that failed during 2005 through 2007.

House wrens and EABL are known to nest multiple times during a breeding season, and it has been suggested that in parts of their range TRES can successfully have two broods. Adult females were captured and banded or recaptured at 91, 98, and 96% of clutches that hatched nestlings among all study sites for HOWR, TRES and EABL, respectively. No female TRES successfully raised two broods in one season, however two females nested successfully after their first nesting attempt had been preyed upon. Multiple nesting attempts by adult females were more common in HOWR (18–29% by year) and EABL (33–56% by year). Despite multiple nesting attempts by female HOWR and EABL, individual nesting attempts were still considered independent samples. Female HOWR and EABL that attempted two clutches within a nesting season began incubating the first brood during the ENS season 95 and 84% of the time, respectively. Female HOWR, TRES and EABL that only attempted one clutch within a nesting season began incubating during the ENS season 47, 62, and 55% of the time, respectively.

Overall, for the reproductive endpoints examined, the populations were successful for the three species studied during the period of 2005 through 2007, but there were some species- and endpoint-specific differences among SAs. Measures of reproductive success were not different for the species examined when compared among years, but were different if separated seasonally into ENS and LNS nest initiation dates. By splitting the nesting season for each studied species into ENS and LNS nesting attempts it was possible to separate the majority of multiple broods (HOWR and EABL) or re-nesting attempts (TRES) and to investigate potential temporal reproductive differences. Clutch size, predicted brood size, predicted number of fledglings, fledgling success, and productivity for HOWR and TRES were greater for ENS nests than for LNS, while hatching success was similar (Table S2). Measures of nesting success for EABL were similar for the ENS and LNS periods.

Mean overall hatching success, fledging success, and productivity for all species varied among years with ranges of 68–88, 54–97, and 48–82%, for HOWR, TRES and EABL, respectively (Table S2). This degree of temporal variation, which was most likely due to seasonal variability, set the range of background variation in these parameters against which effects of PCDD/DF need to be considered. A range of approximately a factor of two seems to be the norm for these parameters as a function of time. Thus, effects due to PCDD/DF would need to be at least a factor of two to be equivalent to the range observed among years, which seems to be due to natural causes. Both ENS and overall predicted brood sizes were greater for HOWR (*F* = 4.03 *p* = 0.0199, *F* = 5.02 *p* = 0.0072, respectively) and TRES (*F* = 5.60 *p* = 0.0049, *F* = 7.80 *p* = 0.0006, respectively) at Saginaw River SAs than at the Tittabawassee River SAs or for TRES at the RAs (Table S2). Fledging success for HOWR LNS (*F* = 5.41 *p* = 0.0053) was greater at RAs despite greater brood sizes (*F* = 4.03 *p* = 0.0199) and predicted number of fledglings (*F* = 4.68 *p* = 0.0108) ENS at Saginaw River SAs (Table S2). Clutch size and predicted number of fledglings were greater for TRES at Saginaw River SAs compared to the other SAs for both the ENS (*F* = 8.55 *p* = 0.0003, *F* = 3.90 *p* = 0.0232, respectively) and overall (*F* = 5.79 *p* = 0.0036, *F* = 5.53 *p* = 0.0047, respectively) time periods (Table S2). Overall productivity for TRES was greater at Saginaw River SAs compared to Tittabawassee River SAs (*F* = 3.96 *p* = 0.0208), while RAs were intermediate (Table S2). Nesting success of EABL was similar between Tittabawassee River SAs and RAs; however Saginaw River SAs were not included in statistical comparisons due to less occupancy.

The majority of HOWR (91%), TRES (80%), and EABL (82%) females bred during only one nesting season. Female TRES at Saginaw River SAs hatched and fledged more nestlings by season (*F* = 9.29 *p* < 0.0001, *F* = 6.83 *p* = 0.0014, respectively) and for the overall study period (*F* = 6.28 *p* = 0.0024, *F* = 5.52 *p* = 0.0049, respectively) than at the other SAs (Table S3). Female HOWR at the Saginaw River SAs hatched more nestlings by season (*F* = 4.05 *p* = 0.0186) than females at Tittabawassee River SAs, while RAs were intermediate (Table S3). Measures of nesting success for EABL were similar between RAs and Tittabawassee River SAs, with individual females fledging up to 13 nestlings over the study period (Table S3).

### Egg and nestling morphometric measurements

Mean masses of eggs by clutch were not different among SAs for HOWR (*F* = 1.89 *p* = 0.1530) or TRES (*F* = 1.15 *p* = 0.3194), but were greater at Tittabawassee River SAs compared to RAs for EABL (*F* = 4.04 *p* = 0.0207). Mean (±SD) masses of eggs for 233 HOWR clutches was 1.45 ± 0.12 g (ranged 1.16–1.80), and was 1.84 ± 0.14 g (ranged 1.49–2.25) for 121 TRES clutches. Mean (±SD) masses of eggs for EABL clutches at Tittabawassee River SAs was 3.12 ± 0.29 g (*n* = 62; ranged 2.37–3.77), at RAs it was 2.96 ± 0.23 g (*n* = 34; ranged 2.47–3.38), while at Saginaw River SAs it was 3.12 ± 0.10 g (*n* = 4; ranged 2.99–3.20). Saginaw River SAs were not included in the among-site comparisons for EABL because of low box occupancy at those locations.

Mean masses of nestlings post-hatch by clutch were similar for HOWR among all SAs, but were different on some days for TRES and EABL. Tree swallow nestlings 8 days post-hatch had greater masses at RAs and Tittabawassee River SAs compared to those at Saginaw River SAs (*F* = 5.52 *p* = 0.0050), while masses on the other days were similar among SAs. Eastern bluebird nestlings had greater masses 8 days (*F* = 14.60 *p* = 0.0003), 12 days (*F* = 13.58 *p* = 0.0004), and 14 days (*F* = 6.08 *p* = 0.0161) post-hatch at Tittabawassee River SAs compared to RAs. Mass gained per day was not different among SAs for HOWR (*F* = 0.79 *p* = 0.4562), TRES (*F* = 0.93 *p* = 0.3965), or EABL (*F* = 3.05 *p* = 0.0853). Mean mass gained per day for all SAs was greatest in EABL (1.87 ± 0.31 g; *n* = 75), least in HOWR (0.97 ± 0.13 g; *n* = 197), and intermediate in TRES (1.66 ± 0.25 g; *n* = 122). 95% confidence intervals of growth rate constants for HOWR broods at RAs, Tittabawassee River SAs, and Saginaw River SAs ranged from 0.40 to 0.52 (*n* = 48), 0.40 to 0.49 (*n* = 123), and 0.40 to 0.56 (*n* = 40), respectively. 95% confidence intervals of growth rate constants for TRES broods at RAs, Tittabawassee River SAs, and Saginaw River SAs ranged from 0.47 to 0.58 (*n* = 39), 0.47 to 0.58 (*n* = 58), and 0.38 to 0.52 (*n* = 37), respectively. 95% confidence intervals of growth rate constants for EABL broods at RAs and Tittabawassee River SAs ranged from 0.29 to 0.45 (*n* = 27) and 0.39 to 0.50 (*n* = 47), respectively. Comparisons were not made between TRES and HOWR mean growth rate constants for locations due to the nearly complete overlap in 95% CIs. However, growth rate constants for EABL (mean ± SD) were similar between RAs (0.37 ± 0.20) and Tittabawassee River SAs (0.44 ± 0.19; *t*_72_ = 1.5453, *p* = 0.1267).

## Discussion

### Nesting success

Nest abandonment and predation events accounted for approximately a quarter of HOWR, TRES, and EABL clutches that failed during 2005 through 2007. In an area of the Hudson River highly contaminated with PCBs, TRES exhibited a greater incidence of abandoned clutches compared to nests upstream of the contamination (McCarty and Secord 1999). However, based solely on empirical comparisons (since statistical analyses were not possible), both abandonment and depredation rates were variable among areas. House sparrows (*Passer domesticus*) and HOWR were the primary avian predators for TRES and EABL nests, while HOWR nests were depredated primarily by mammals. These events are natural occurrences that affect bird populations and are part of the experiment when conducting field studies. Since the number of abandonments was variable among areas, this was not likely related to exposure to PCDD/DFs. As for predation, while it cannot be definitively related to effects of exposure to PCDD/DFs, potential effects on behavior related to protecting the nests cannot be definitively eliminated. However, since no empirical differences were observed in nest attentiveness (defined as the number of feeding visits by adults during 30 min nest observations; data not presented) and the fact that these species of birds have no capability to defend the nest against these types of predation events, it is unlikely that the differences in rates of predation among areas were related to effects of PCDD/DFs.

Fledging success for HOWR was less for all SAs during the LNS period compared to the ENS, however it was 43% less at Saginaw River SAs compared to only 12 and 20% at RAs and Tittabawassee River SAs, respectively. Similarly, fledging success for HOWR in Wyoming was less for those breeding later in the season (Finch 1991), while fledging success in Michigan was similar between defined temporal periods (Neigh et al. 2007). It is unknown why broods at Saginaw River SAs had more than two-fold less LNS fledging success compared to the other SAs. Despite the lesser fledging success for LNS nesting HOWR at Saginaw River SAs, the overall predicted brood size was greater and the overall predicted number of fledglings was similar to other SAs.

Reproductive success of TRES nestlings based on counts (clutch size, predicted brood size and predicted number of fledglings) were greater for ENS and for overall nesting attempts at Saginaw River SAs compared to the other SAs. The inherent interrelatedness of these variables undoubtedly is the reason for the similar statistical trends. The difference was primarily due to the reproductive output of TRES breeding at S-9, and likely related to site-specific differences in availability of food or habitat between it and the other sites. Most boxes at S-9 were adjacent to the bank opposed to other study sites in which the boxes were separated from the bank by floodplain forest. While quantification of food availability was beyond the scope of this project, the relative abundance of Diptera and Hemiptera in close proximity to the nest boxes was potentially greater at this site (Fredricks et al. 2011a). Sites in Ontario had a 7-fold difference in insect abundance which resulted in differential productivity of TRES among sites (Quinney et al. 1986). Also, TRES breeding downstream of a pulp/paper mill also exhibited greater reproductive output likely due to nutrient enrichment and a subsequent increase in abundance of insects (Wayland et al. 1998). Greater abundance of food early in the nesting season at study locations along a lakeshore compared to along a roadside resulted in larger TRES clutches (Dunn and Hannon 1992). However, in the current study differences among habitat characteristics were less drastic among SAs. Natural variation in availability of food and subsequent effects upon reproductive output defines the range of natural variability in parameters against which effects of PCDD/DF need to be considered.

For all species studied, hatching success was the only nesting success endpoint measured that was similar between ENS and LNS nesting attempts. Hatching success is used as one of the most sensitive endpoints for avian exposure assessment (Custer et al. 2003, 2005; Nosek et al. 1992, 1993; Powell et al. 1996; Thiel et al. 1988). It was predicted that increased time spent on-site would lead to greater site-specific exposure to contaminants, which would result in decreased hatching success later in the season. However, like hatching success, egg concentrations of ΣPCDD/DF TEQs_WHO-Avian_ and ΣPCDD/DFs were similar throughout the breeding season (Fredricks et al. 2011d), which was likely due to the fact that most passerine species ingest resources for egg production near the time of breeding (Drent and Daan 1980; Perrins 2008; Winkler and Allen 1996).

For HOWR and TRES, clutch size, predicted brood size, predicted number of fledglings, fledging success, and productivity were greater for ENS than for LNS nesting attempts, while for EABL these endpoints were similar. Several studies have documented lesser sizes of clutches later in the breeding season (Kennedy and Power 1990; Kennedy and White 1991; Neigh et al. 2007; Perrins and McCleery 1989; Robinson and Rotenberry 1991; Stutchbury and Robertson 1985), which inherently should lead to lesser predicted brood sizes and predicted number of fledglings. Poorer reproductive success later in the breeding season has been previously reported for several species (DeSteven 1978; Lombardo 1994; Perrins and McCleery 1989; Pinkowski 1979; Stutchbury and Robertson 1988). The specific cause(s) of the reduced rates of reproductive success across SAs for these endpoints during LNS nesting attempts is beyond the scope of this project. However, since these endpoints generally decreased similarly across SAs and concentrations of PCDD/DFs in eggs and nestlings were similar throughout the nesting season, it was unlikely due to greater exposure to contaminants at downstream SAs.

Site-specific exposures of passerine birds to dioxin-like compounds throughout the United States and Canada have reported mixed results as to potential reproductive effects. The majority of studies have reported variable or no significant reproductive effects (Bishop et al. 1999; Harris and Elliott 2000; McCarty and Secord 1999; Neigh et al. 2006b, 2007; Thiel et al. 1988), while a significant negative correlation was observed for TRES hatching success and egg concentrations on the primarily PCB-contaminated Housatonic River in Massachusetts (Custer et al. 2003) and site-specific hatching success was less on the primarily TCDD-contaminated Woonasquatucket River in Rhode Island (Custer et al. 2005). However, studied species along the Tittabawassee River had similar ΣPCDD/DF or TEQ_WHO-Avian_ concentrations in eggs (Fredricks et al. 2011d) to the studies on the Housatonic and Woonasquatucket rivers. In this study a similar effect was not observed in hatching success based on site-specific comparisons. It is important to note that a site-specific difference in reproductive success endpoints related to TRES eggs (clutch size and hatching success) would not be expected based on the similar concentrations of ΣPCDD/DFs or TEQs_WHO-Avian_ in eggs between RAs and SAs (Fredricks et al. 2011d). Conversely, a significant logistic regression of hatching success and ΣPCDD/DF TEQ_WHO-Avian_ concentrations in TRES eggs from the Tittabawassee River SAs was reported (Fredricks et al. 2011c) based on individual eggs from clutches with associated hatching success measurements. For nesting success endpoints, measured values were within the species-specific ranges previously reported in the literature (Bauldry et al. 1995; Finch 1989; Neigh et al. 2006b; 2007; Pinkowski 1979). In this study, differences in nesting success in this study between ENS and LNS indicate that it can be useful to consider an earlier and later reproductive period when investigating the potential for effects of environmental stressors such as contaminants. Separating the reproductive periods in this way can reduce overall variability against which the effects of contaminants need to be considered and increase the overall power of the study to discriminate effects of contaminants relative to the natural variability observed in wild populations of birds among locations and between earlier and later periods of reproduction.

Incidences of deformities and embryo death have been correlated with colonial water birds in the Great Lakes from 1986 to 1991, where they were exposed to planar halogenated compounds, such as the PCDD/DFs (Ludwig et al. 1996). Deformities of the lower bill and subcutaneous edema were documented in a single clutch of wood duck (*Aix sponsa*) eggs exposed to PCDD/DFs in Arkansas (White and Seginak 1994). Developmental abnormalities were also observed after *in ovo* exposure to TCDD and other dioxin-like compounds in various colonial, fish-eating water birds, and included edemas of the head and neck, liver damage, and skeletal and beak deformities (Blankenship et al. 2003; Hoffman et al. 1998; Perrins 2008), however similar abnormalities were not always present (Nosek et al. 1993). Several studies of sites contaminated with either PCBs or PCDD/DFs throughout the US on HOWR, TRES and EABL have not reported developmental abnormalities (Custer et al. 2003, 2005; Neigh et al. 2006b, 2007). During routine monitoring of nests, two TRES nestlings from a clutch at R-1 had foot deformities. One had a club foot and the other was missing a foot. These two nestlings with abnormal legs survived through fledge, and it is unknown whether the potential deformities were related to with site-specific contaminant exposures. No other deformities were observed for EABL or HOWR among all SAs for the duration of the study. The fact that the passerines studied here did not exhibit deformities that were related to exposure to similar or greater TEQ_WHO-Avian_ concentrations (Fredricks et al. 2011d) than the colonial water birds of the Great Lakes could be due to the rare nature of these deformities even in highly exposed populations or due to species-specific differences in the sensitivity of birds to dioxin-like compounds (Karchner et al. 2006).

### Egg and nestling morphometric measurements

Differences in nestling condition or masses of eggs have been investigated for possible effects on nest productivity with mixed results (Robinson and Rotenberry 1991; Wiggins 1990). Stresses such as exposure to dioxin-like compounds can alter energetics that can lead to reduced nestling growth rates. Tree swallows exposed to oil sands mining wastes had lesser masses compared to unexposed populations (Gentes et al. 2006). However, TRES nestlings exposed to PCBs on the Hudson River in New York had similar or greater growth compared to upstream locations without PCB contamination (McCarty and Secord 1999). Mean masses of TRES eggs were greater at PCB contaminated versus reference sites along the Kalamazoo River in Michigan (Neigh et al. 2006b). Egg and nestling masses from the current study are within the ranges presented for HOWR (Neigh et al. 2007), TRES (DeSteven 1978; Harris and Elliott 2000; Neigh et al. 2006b), and EABL (Neigh et al. 2007). Despite slight differences in masses of TRES nestlings and EABL eggs and nestlings for some measurements, the overall trend was similar between SAs for egg masses, nesting masses, nestling mass gained/day, and growth rate constants.

Researchers have monitored birds exposed to contaminants for molecular (Custer et al. 2005, 2001; Papp et al. 2005), immune (Dods et al. 2005; Martinovic et al. 2003; Mayne et al. 2004), morphometric (Custer et al. 2005; DeWitt et al. 2006; Henshel et al. 1997), and genetic (Stapleton et al. 2001) responses with mixed results. The predominant limitation of these measurement endpoints is relating them to altered survival or reproductive performance in field studies, while similar measurement endpoints are useful in laboratory studies to determine dose–response relationships (Head and Kennedy 2007). Variability inherent in field studies that is generally assumed to be similar between proximally located exposed and unexposed sites, such as habitat, weather, and genetic relatedness of adults combined with limited sample sizes can add enough uncertainty to mask trends for these response variables. As a result the current study focused on collecting data on response variables at the population level opposed to individual-based responses.

## Conclusions

Overall reproductive success of HOWR, TRES and EABL nesting in the river floodplains near Midland, Michigan was investigated to determine if these parameters were adversely affected by the greater concentrations of ΣPCDD/DFs in sediments and biota at downstream SAs. Despite sediment and biota TEQ_WHO-Avian_ concentrations comparable to some of the most contaminated sites currently known throughout North America, passerines breeding along the Tittabawassee and Saginaw rivers brooded successful nests. Hatching success, a measurement endpoint that has been shown in both field and laboratory studies to be negatively impacted by exposure to dioxin-like compounds, was similar among all SAs based on site-specific comparisons. Hatching success was also the only variable that was temporally consistent for all species, while the majority of other measurement endpoints quantified for HOWR and TRES were greater earlier in the breeding season. Tree swallows at S-9 had greater values for egg and nestling count variables compared to the other SAs, which could be due to habitat differences and/or greater invertebrate abundance at this location. In general, reproductive success was similar or greater at downstream SAs during 2005–2007. Other manuscripts present detailed information and discuss the implications of these results by incorporating data from tissue exposure (Fredricks et al. 2011d) and dietary exposure (Fredricks et al. 2011a) into aquatic (Fredricks et al. 2011c) and terrestrial (Fredricks et al. 2011b) passerine risk assessments.

## Electronic supplementary material

Below is the link to the electronic supplementary material.

Supplementary material 1 (PDF 70 kb) **Figure S1** Frequency of clutch incubation initiations for house wren (black), tree swallow (open), and eastern bluebird (checked) clutches during 7-d windows for a) 2005, b) 2006, and c) 2007 for all study sites near Midland, Michigan, USA. Grey-topped bars indicate a known subsequent nesting attempt by a female during that season. Scale varies between years

Supplementary material 2 (PDF 178 kb)
