# Supplementary material for: Reproductive success of three passerine species exposed to dioxin-like compounds near Midland, Michigan, USA
Source: Ecotoxicology. 2012 Mar 6;21(4):1145–54. doi: 10.1007/s10646-012-0869-4 (PMC3325409; doi:10.1007/s10646-012-0869-4)
Supplement: Supplementary file 1 — Supplementary material 1 (PDF 70 kb) Figure S1 Frequency of clutch incubation initiations for house wren (black), tree swallow (open), and eastern bluebird (checked) clutches during 7-d windows for a) 2005, b) 2006, and c) 2007 for all study sites near Midland, Michigan, USA. Grey-topped bars indicate a known subsequent nesting attempt by a female during that season. Scale varies between years [file 10646_2012_869_MOESM1_ESM.pdf]

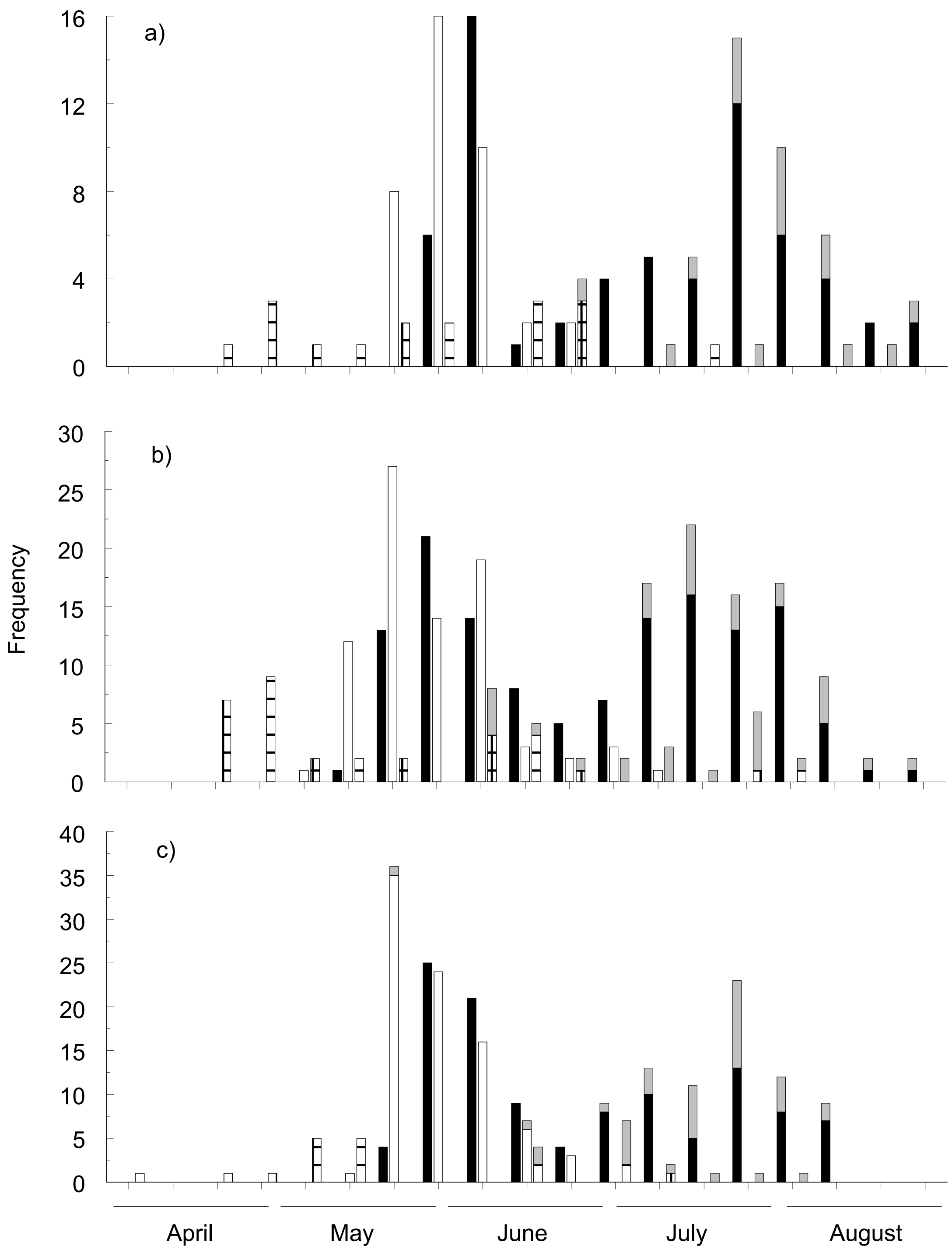

Figure S1. Frequency of clutch incubation initiations for house wren (black), tree swallow (open), and eastern bluebird (checked) clutches during 7-d windows for a) 2005, b) 2006, and c) 2007 for all study sites near Midland, Michigan, USA. Grey-topped bars indicate a known subsequent nesting attempt by a female during that season. Scale varies between years.
