# Supplementary material for: Reproductive success of three passerine species exposed to dioxin-like compounds near Midland, Michigan, USA
Source: Ecotoxicology. 2012 Mar 6;21(4):1145–54. doi: 10.1007/s10646-012-0869-4 (PMC3325409; doi:10.1007/s10646-012-0869-4)
Supplement: Supplementary file 2 — Supplementary material 2 (PDF 178 kb) [file 10646_2012_869_MOESM2_ESM.pdf]

Table S1. Nesting attempt outcomes and percentages of initiated clutches for house wrens, tree swallows, and eastern bluebirds nesting in the Chippewa, Tittabawassee, and Saginaw River floodplains, Midland, Michigan, USA.

	2005		2006			2007			Overall
	R-1 to R-2	T-3 to T-6	R-1 to R-2	T-3 to T-6	S-7 and S-9[a]	R-1 to R-2	T-3 to T-6	S-7 and S-9	
**House wren**									
Initiated[b]	24	75	36	110	27	36	86	33	427
Incubated[c]	19 (79%)	60 (80%)	34 (94%)	95 (86%)	26 (96%)	35 (97%)	75 (87%)	31 (94%)	375 (88%)
Hatched[d]	19 (79%)	60 (80%)	32 (89%)	87 (79%)	26 (96%)	34 (94%)	64 (74%)	29 (88%)	351 (82%)
Fledged[e]	15 (63%)	43 (57%)	25 (69%)	70 (64%)	18 (67%)	31 (86%)	56 (65%)	19 (58%)	277 (65%)
Predated[f]	0 (0%)	11 (15%)[j]	7 (19%)	13 (12%)	3 (11%)[j]	2 (6%)	24 (28%)[k]	11 (33%)[k]	71 (17%)
Abandoned[g]	4 (17%)	8 (11%)	0 (0%)	10 (9%)	3 (11%)	2 (6%)	5 (6%)	3 (9%)	35 (8%)
Other[h]	3 (13%)	10 (13%)	3 (9%)	12 (11%)	1 (4%)	0 (0%)	1 (1%)	0 (0%)	30 (7%)
Failed[i]	2 (8%)	3 (4%)	1 (3%)	5 (5%)	2 (7%)	1 (3%)	0 (0%)	0 (0%)	14 (3%)

**Tree swallow**									
Initiated	24	29	26	38	31	25	48	24	245
Incubated	14 (58%)	24 (83%)	23 (88%)	34 (89%)	28 (90%)	23 (92%)	43 (90%)	23 (96%)	212 (87%)
Hatched	14 (58%)	23 (79%)	21 (81%)	30 (79%)	26 (84%)	21 (84%)	38 (79%)	23 (96%)	196 (80%)
Fledged	14 (58%)	18 (62%)	19 (73%)	26 (68%)	24 (77%)	18 (72%)	34 (71%)	23 (96%)	176 (72%)
Predated	2 (8%)	9 (31%)[l]	2 (8%)	7 (18%)	3 (10%)	4 (16%)	10 (21%)[j]	1 (4%)	38 (16%)
Abandoned	7 (29%)	0 (0%)	2 (8%)	3 (8%)	1 (3%)	2 (8%)	1 (2%)	0 (0%)	16 (7%)
Other	1 (4%)	0 (0%)	3 (12%)	1 (3%)	2 (6%)	1 (4%)	3 (6%)	0 (0%)	11 (5%)
Failed	0 (0%)	2 (7%)	0 (0%)	1 (3%)	1 (3%)	0 (0%)	0 (0%)	0 (0%)	4 (2%)
**Eastern bluebird**									
Initiated	12	18	15	40	5	14	18	0[m]	122
Incubated	10 (83%)	15 (83%)	15 (100%)	33 (83%)	4 (80%)	12 (86%)	17 (94%)		106 (87%)
Hatched	10 (83%)	15 (83%)	12 (80%)	29 (73%)	4 (80%)	10 (71%)	16 (89%)		96 (79%)
Fledged	7 (58%)	11 (61%)	10 (67%)	20 (50%)	2 (40%)	9 (64%)	16 (89%)		75 (61%)

Predated	1 (8%)	3 (17%)[j]	1 (7%)[f]	7 (18%)[g]	2 (40%)	3 (21%)	2 (11%)		19 (16%)
Abandoned	2 (17%)	2 (11%)	4 (27%)	5 (13%)	0 (0%)	0 (0%)	0 (0%)		13 (11%)
Other	1 (8%)	2 (11%)	0 (0%)	8 (20%)	1 (20%)	2 (14%)	0 (0%)		14 (11%)
Failed	1 (8%)	0 (0%)	0 (0%)	0 (0%)	0 (0%)	0 (0%)	0 (0%)		1 (1%)

[a] S-7 and S-9 were monitored in 2006 and 2007

[b] Nests in which at least one egg was laid

[c] Complete clutches that were warm to the touch on subsequent days

[d] Nests that successfully hatched eggs

[e] Fledged at least one nestling

[f] Nests that were disturbed or had signs of predator access to the box

[g] Nest with eggs and/or nestlings present but without an adult present for at least 7 days

[h] Nests failure was caused by human interference or undeterminable reasons

[i] Failed nesting attempts that aren’t included in the other categories (predated, abandoned, or other). The sum of all unsuccessful nesting attempts (predated, abandoned, other, and failed) is equal to the difference between initiated nesting attempts and those that successfully fledged a nestling.

[j] One nest that was preyed upon successfully fledged at least one nestling

[k] Two nests that were preyed upon successfully fledged at least one nestling

[l] Three nests that were preyed upon successfully fledged at least one nestling

[m] No clutches were initiated at S-7 and S-9 in 2007

Table S2. Measures of nesting success for ENS, LNS, and all nesting attempts for house wrens, tree swallows, and eastern bluebirds breeding in the river floodplains near Midland, Michigan during 2005-2007.

	ENS nesting attempts						LNS nesting attempts						All nesting attempts					
	R-1 to R-2		T-3 to T-6		S-7 and S-9[b]		R-1 to R-2		T-3 to T-6		S-7 and S-9		R-1 to R-2		T-3 to T-6		S-7 and S-9	
	n	Mean (SD)[c]	*n*	Mean (SD)	*n*	Mean (SD)	*n*	Mean (SD)	*n*	Mean (SD)	*n*	Mean (SD)	*n*	Mean (SD)	*n*	Mean (SD)	*n*	Mean (SD)
**House wren**																		
Hatching Success[d]	38	0.81 (0.24)	93	0.76 (0.25)	23	0.83 (0.26)	44	0.80 (0.19)	113	0.78 (0.23)	30	0.81 (0.20)	82	0.81 (0.21)	206	0.77 (0.24)	53	0.82 (0.23)
Fledging Success[e]	35	0.93 (0.19)	84	0.93 (0.18)	20	0.97 (0.071)	42	0.81[A] (0.34)	106	0.73[A] (0.40)	25	0.54[B] (0.42)	77	0.86[A] (0.29)	190	0.82[AB] (0.33)	45	0.73[B] (0.38)
Productivity[f]	35	0.78 (0.25)	84	0.73 (0.23)	20	0.85 (0.21)	42	0.65 (0.32)	106	0.61 (0.37)	25	0.48 (0.40)	77	0.71 (0.29)	190	0.66 (0.32)	45	0.65 (0.37)
Clutch Size	38	6.4 (0.86)	93	6.0 (1.3)	23	6.3 (1.6)	45	5.7 (0.90)	113	5.3 (1.0)	30	5.6 (1.2)	83	6.0 (0.96)	206	5.6 (1.2)	53	5.9 (1.4)

Predicted		5.5[AB]		4.9[B]		5.8[A]		4.6		4.2		4.6		5.0[AB]		4.5[B]		5.1[A]
Brood Size[g]	35	(1.6)	84	(1.6)	20	(1.5)	42	(1.3)	106	(1.4)	25	(1.5)	77	(1.5)	190	(1.6)	45	(1.6)
Predicted #		5.1[AB]		4.5[B]		5.7[A]		3.8		3.2		2.8		4.4		3.8		4.1
Fledglings[h]	35	(1.9)	84	(1.8)	20	(1.5)	42	(1.9)	106	(2.1)	25	(2.5)	77	(2.0)	190	(2.1)	45	(2.5)
**Tree swallow**																		
Hatching		0.82		0.78		0.87		0.80		0.73		0.84		0.81		0.76		0.86
Success	34	(0.22)	49	(0.22)	30	(0.17)	20	(0.24)	37	(0.27)	19	(0.27)	54	(0.23)	86	(0.25)	49	(0.21)
Fledging		0.95		0.95		0.95		0.96		0.85		0.87		0.95		0.91		0.92
Success	33	(0.18)	48	(0.14)	30	(0.14)	19	(0.11)	33	(0.30)	17	(0.22)	52	(0.16)	81	(0.22)	47	(0.17)
		0.80		0.75		0.82		0.78		0.63		0.79		0.80[AB]		0.70[B]		0.81[A]
Productivity	33	(0.23)	48	(0.21)	30	(0.19)	19	(0.23)	33	(0.30)	17	(0.27)	52	(0.23)	81	(0.26)	47	(0.22)
		5.1[B]		5.4[B]		6.0[A]		4.8		4.9		4.9		5.0[B]		5.2[B]		5.6[A]
Clutch Size	35	(1.0)	51	(0.94)	31	(0.82)	21	(1.1)	38	(0.78)	19	(1.0)	56	(1.1)	89	(0.90)	50	(1.0)
Predicted		4.3[B]		4.3[B]		5.3[A]		4.0		3.6		4.5		4.2[B]		4.0[B]		5.0[A]
Brood Size	33	(1.3)	48	(1.2)	30	(1.3)	19	(1.5)	33	(1.4)	17	(1.3)	52	(1.4)	81	(1.4)	47	(1.4)
Predicted #	33	4.1[AB]	48	4.1[B]	30	5.0[A]	19	3.8	33	3.1	17	4.0	52	4.0[AB]	81	3.7[B]	47	4.6[A]

Fledglings		(1.5)		(1.4)		(1.4)		(1.4)		(1.6)		(1.7)		(1.5)		(1.5)		(1.6)
**Eastern bluebird**[i]																		
Hatching Success	15	0.72 (0.31)	23	0.78 (0.29)	1	1.0	16	0.68 (0.38)	29	0.88 (0.17)	1	0.67	31	0.70 (0.34)	52	0.84 (0.24)	2	0.83 (0.24)
Fledging Success	14	0.80 (0.33)	21	0.84 (0.25)	1	0.80	14	0.88 (0.29)	28	0.89 (0.24)	1	1.0	28	0.84 (0.31)	49	0.87 (0.24)	2	0.90 (0.14)
Productivity	14	0.61 (0.33)	21	0.72 (0.24)	1	0.80	14	0.65 (0.33)	28	0.79 (0.27)	1	0.67	28	0.63 (0.33)	49	0.76 (0.26)	2	0.73 (0.094)
Clutch Size	16	4.8 (0.86)	27	4.8 (0.64)	2	5.0	17	4.4 (1.0)	30	4.4 (0.81)	2	3.0	33	4.5 (0.94)	57	4.6 (0.75)	4	4.0 (1.2)
Predicted Brood Size	14	3.7 (1.4)	21	4 (0.89)	1	5.0	14	3.6 (1.5)	28	3.9 (1.1)	1	2.0	28	3.6 (1.4)	49	4.0 (1.0)	2	3.5 (2.1)
Predicted # Fledglings	14	2.9 (1.7)	21	3.4 (1.2)	1	4.0	14	3.1 (1.6)	28	3.6 (1.4)	1	2.0	28	3.0 (1.6)	49	3.5 (1.3)	2	3.0 (1.4)

[a] ENS = early nesting season (nests incubated prior to June 23, May 24, and May 29 for HOWR, TRES, and EABL, respectively) and LNS = late nesting season (nests initiated after those species specific dates)

[b] S-7 and S-9 were monitored in 2006 and 2007

[c] Statistical comparisons were made for all endpoints however only endpoints that were statistically different (p<0.05) are indicated with different uppercase letters

[d] Hatching success was adjusted for any eggs removed for contaminant analyses or broken by researchers

[e] Fledging success includes nestlings collected for contaminant analyses if the remainder of the clutch was successful

[f] Productivity is defined as the number of nestlings fledged per eggs laid

[g] Brood size was predicted based on clutch size and hatching success

[h] Predicted number of fledglings was defined as the product of clutch size and productivity

[i] S-7 and S-9 were not included in statistical comparisons for eastern bluebirds

Table S3. Mean overall measures of nesting success by study area for individually identified female house wrens, tree swallows, and eastern bluebirds breeding in the river floodplains near Midland, Michigan. Experimental units for individual seasons are unique females per year and for the overall study are unique females.

	Individual seasons						Overall study					
	R-1 to R-2		T-3 to T-6		S-7 and S-9[a]		R-1 to R-2		T-3 to T-6		S-7 and S-9	
	n	Mean ± SD (range)[b]	*n*	Mean ± SD (range)	*n*	Mean ± SD (range)	*n*	Mean ± SD (range)	*n*	Mean ± SD (range)	*n*	Mean ± SD (range)
**House wren**												
Nesting attempts	58	1.3 ± 0.46 (1–2)	155	1.2 ± 0.40 (1–2)	36	1.3 ± 0.51 (1–3)	52	1.4 ± 0.67 (1–4)	142	1.3 ± 0.57 (1–4)	34	1.4 ± 0.60 (1–3)
Eggs laid	58	7.8 ± 3.4 (4–15)	155	6.7 ± 2.6 (2–14)	36	7.8 ± 3.1 (4–16)	52	8.7 ± 4.8 (4–25)	142	7.3 ± 3.4 (2–26)	34	8.2 ± 3.8 (4–21)
Nestlings hatched	58	6.2 ± 3.4[AB] (2–14)	155	5.2 ± 2.3[B] (1–13)	36	6.5 ± 2.9[A] (3–14)	52	6.9 ± 4.5 (2–23)	142	5.6 ± 3.0 (1–26)	34	6.9 ± 3.5 (3–18)
Nestlings fledged	58	5.5 ± 3.6 (0–14)	151	4.4 ± 2.4 (0–13)	36	5.1 ± 3.4 (0–13)	52	6.1 ± 4.6 (0–22)	139	4.8 ± 3.0 (0–25)	34	5.4 ± 4.0 (0–17)

**Tree swallow**												
Nesting attempts	60	1.0 ± 0.0 (1–1)	88	1.0 ± 0.15 (1–2)	49	1.0 ± 0.0 (1–1)	51	1.2 ± 0.42 (1–3)	72	1.3 ± 0.53 (1–3)	38	1.3 ± 0.46 (1–2)
Eggs laid	60	4.8 ± 1.2^{B} (1–7)	88	5.4 ± 1.4^{AB} (1–13)	49	5.6 ± 1.0^{A} (3–7)	51	5.5 ± 2.5^{B} (1–15)	72	6.6 ± 3.2^{AB} (1–20)	38	7.2 ± 2.9^{A} (3–14)
Nestlings hatched	60	3.9 ± 1.4^{B} (1–6)	88	3.8 ± 1.4^{B} (0–7)	49	4.8 ± 1.4^{A} (1–7)	51	4.5 ± 2.1^{B} (1–11)	72	4.7 ± 2.7^{B} (0–15)	38	6.2 ± 2.9^{A} (1–13)
Nestlings fledged	54	3.6 ± 1.5^{B} (0–6)	85	3.4 ± 1.6^{B} (0–7)	47	4.4 ± 1.6^{A} (1–7)	51	4.2 ± 2.3^{B} (0–11)	69	4.2 ± 2.8^{B} (0–13)	36	5.7 ± 2.8^{A} (1–13)
**Eastern bluebird**[c]												
Nesting attempts	21	1.5 ± 0.68 (1–3)	37	1.6 ± 0.59 (1–3)	2	(2–2)	18	1.7 ± 1.3 (1–6)	30	2.0 ± 1.2 (1–5)	2	(2–2)
Eggs laid	21	6.8 ± 3.6 (2–15)	37	7.3 ± 3.1 (2–16)	2	(8–8)	18	7.9 ± 6.6 (2–29)	30	9.0 ± 5.9 (2–24)	2	(8–8)

Nestlings hatched	21	4.9 ± 3.1 (0–13)	37	5.9 ± 2.5 (2–11)	2	(6–7)	18	5.7 ± 4.9 (0–20)	30	7.4 ± 4.4 (2–18)	2	(6–7)
Nestlings fledged	20	4.3 ± 2.4 (0–10)	35	4.5 ± 2.5 (0–10)	2	(2–4)	17	5.1 ± 3.8 (0–13)	28	5.7 ± 3.4 (0–13)	2	(2–4)
Male nestlings	19	2.3 ± 1.5 (0–6)	35	2.8 ± 1.5 (0–7)	2	(1–3)	16	2.6 ± 2.2 (0–8)	28	3.5 ± 2.1 (0–8)	2	(1–3)
Female nestlings	17	2.6 ± 2.2 (0–8)	31	2.3 ± 1.4 (1–5)	2	(1–1)	15	2.9 ± 2.9 (0–10)	25	2.8 ± 1.7 (1–7)	2	(1–1)

[a] S-7 and S-9 were monitored in 2006 and 2007

[b] Means with different uppercase letters were significantly different ($p<0.05$)

[c] S-7 and S-9 were not included in statistical comparisons for eastern bluebirds
